# Investigating harms of testing for ovarian cancer – psychological outcomes and cancer conversion rates in women with symptoms of ovarian cancer: A cohort study embedded in the multicentre ROCkeTS prospective diagnostic study

**DOI:** 10.1111/1471-0528.17813

**Published:** 2024-03-31

**Authors:** Fong Lien Kwong, Caroline Kristunas, Clare Davenport, Ridhi Aggarwal, Jon Deeks, Sue Mallett, Sean Kehoe, Dirk Timmerman, Tom Bourne, Hilary Stobart, Richard Neal, Usha Menon, Alex Gentry-Maharaj, Lauren Sturdy, Ryan Ottridge, Sudha Sundar

**Affiliations:** 1 Institute of Applied Health Research, University of Birmingham, Birmingham, UK; 2 Pan Birmingham Gynaecological Cancer Centre, City Hospital, Birmingham, UK; 3 Institute of Cancer and Genomic Sciences, University of Birmingham, Birmingham, UK; 4 Centre for Medical Imaging, University College London, London, UK; 5 St Peter’s College, University of Oxford, Oxford, UK; 6 Department of Development and Regeneration, KU Leuven, Leuven, Belgium; 7 Department of Obstetrics and Gynaecology, University Hospitals KU Leuven, Leuven, Belgium; 8 Department of Metabolism, Digestion and Reproduction, Faculty of Medicine, Imperial College London, London, UK; 9 Patient Representative, Birmingham, UK; 10 University of Exeter Medical School, University of Exeter, Exeter, UK; 11 Institute of Clinical Trials and Methodology, University College London, London, UK

**Keywords:** anxiety, conversion rates, depression, diagnosis, fast-track pathways, ovarian cancer

## Abstract

**Objective:**

To investigate psychological correlates in women referred with suspected ovarian cancer via the fast-track pathway, explore how anxiety and distress levels change at 12 months post-testing, and report cancer conversion rates by age and referral pathway.

**Design:**

Single-arm prospective cohort study.

**Setting:**

Multicentre. Secondary care including outpatient clinics and emergency admissions.

**Population:**

A cohort of 2596 newly presenting symptomatic women with a raised CA125 level, abnormal imaging or both.

**Methods:**

Women completed anxiety and distress questionnaires at recruitment and at 12 months for those who had not undergone surgery or a biopsy within 3 months of recruitment.

**Main outcome measures:**

Anxiety and distress levels measured using a six-item short form of the State–Trait Anxiety Inventory (STAI-6) and the Impact of Event Scale – Revised (IES-r) questionnaire. Ovarian cancer (OC) conversion rates by age, menopausal status and referral pathway.

**Results:**

Overall, 1355/2596 (52.1%) and 1781/2596 (68.6%) experienced moderate-to-severe distress and anxiety, respectively, at recruitment. Younger age and emergency presentations had higher distress levels. The clinical category for anxiety and distress remained unchanged/worsened in 76% of respondents at 12 months, despite a non-cancer diagnosis. The OC rates by age were 1.6% (95% CI 0.5%–5.9%) for age <40 years and 10.9% (95% CI 8.7%–13.6%) for age ≥40 years. In women referred through fast-track pathways, 3.3% (95% CI 1.9%–5.7%) of pre- and 18.5% (95% CI 16.1%–21.0%) of postmenopausal women were diagnosed with OC.

**Conclusions:**

Women undergoing diagnostic testing display severe anxiety and distress. Younger women are especially vulnerable and should be targeted for support. Women under the age of 40 years have low conversion rates and we advocate reducing testing in this group to reduce the harms of testing.

## 1 Introduction

Ovarian cancer (OC), defined as ovarian, fallopian tube and primary peritoneal carcinomas, is the sixth most common cancer in women in the UK.^1^ Disease stage at presentation is a robust predictor of survival. Most (95%) women diagnosed with stage 1 OC survive their disease for 5 years, compared with 15% of those diagnosed with stage 4 disease. Screening has not shown a significant reduction in mortality,^2,3^ and national guidelines recommend that women with symptoms should undergo sequential testing with CA125 and ultrasound scan.^4^ Those with a raised CA125 level and abnormal imaging should be referred through a fast-track pathway to specialist gynaecologists within 2 weeks. Nearly one in three women with OC in the UK were diagnosed through this pathway in 2013, whereas one in four cases presented via emergency routes.^5^ Unfortunately, the non-specific nature of the symptoms together with the low sensitivity and specificity of CA125 result in a high rate of false-positive referrals. In fact, only 4.0% (7978 of 198 783) of women who were urgently referred with a suspected gynaecological cancer, including OC, in 2020–2021 had a confirmed diagnosis, representing a cancer conversion rate of 4.0%.^6^

The harms of cancer testing including its therapeutic, financial, legal and psychosocial implications are well described.^7^ In certain cancer subtypes, namely breast, lung, colorectal and cervical cancers,^8–11^ where population-based testing has demonstrated a clear mortality benefit, the survival benefit following an early diagnosis undoubtedly out-weighs its psychological harms. For OC, however, screening has not been shown to confer any survival advantage and the benefits of diagnostic testing through the fast-track pathway remain unclear. The harms of OC testing should thus be carefully considered, especially in premenopausal women where the incidence of an ovarian cyst being cancerous is one in 1000, compared with three in 1000 in those aged ≥50 years.^12^

Our previous review highlighted that most studies reporting on the psychological harms of OC testing were conducted in women attending for screening and not diagnostic testing.^13^ More recent studies have described the patient experience following a referral via the fast-track pathway for OC.^14–17^ All these studies were qualitative, with a sample size ranging from 24 to 417 participants and with patient experiences evaluated at a single time point. In this study, we report on anxiety and distress levels at two time points in over 2500 participants, identify which variables correlated with psychological harms, and analyse the OC conversion rate by age, menopausal status and referral pathway.

## 2 Methods

### 2.1 Patient and public involvement in the research

The patient information leaflet for the Refining Ovarian Cancer Test Accuracy Scores (ROCkeTS) study was reviewed by four research advocates from the Target Ovarian Cancer charity. A patient representative sat on the project management group and regularly advised on study conduct.

### 2.2 Variables under investigation

We had previously performed a systematic review to identify which patient and/or organisational factors may modify the psychological impact of cancer testing.^13^ Based on clinical experience, we also hypothesised that women with risk factors for OC or with existing or previous gynaecological complaints may be self-aware about their higher risk of OC from publicly available information platforms, such as charity leaflets.^18–20^

### 2.3 Study protocol

ROCkeTS is a single-arm prospective observational diagnostic test accuracy study, whereby all participants donate a blood sample for Risk of Malignancy Algorithm (ROMA) biomarker testing,^21^ which predicts the likelihood of an ovarian malignancy based on the results for CA125 and HE4 and menopausal status, and undergo an ultrasound scan, scored using International Ovarian Tumour Analysis terminology.^22^ Participants completed a series of questionnaires at various time points (Figure S1). Women were recruited from 24 hospitals across the UK in outpatient settings (fast-track pathway, non-urgent outpatient referrals) and following emergency admissions. All participants were given an information leaflet and had their eligibility confirmed by a doctor. Written informed consent was obtained from all participants. Patient data were collected on standard pro formas. The outcome of testing was ascertained by histology from biopsy or surgery, if these were performed within 3 months of referral, or at clinical follow up at 12 months using a well-being questionnaire for those who had not undergone a biopsy or surgery. The results for the diagnostic test accuracy of the biomarkers and ultrasound testing are not yet available.

### 2.4 Participants

Women aged between 16 and 90 years who had been referred to secondary care with symptoms and a raised CA125 level, an abnormal scan or both were included. Pregnant women or those with a previous history of OC were excluded. Postmenopausal recruitment commenced in June 2015 and was completed in March 2018. Premenopausal recruitment commenced in June 2015 and was completed in March 2023. After March 2018 (protocol version 7.0), premenopausal women were only eligible if they were scheduled to undergo a procedure (surgery or biopsy) because of the very low rates of OC in premenopausal recruits. Only women recruited prior to the protocol change were included in the calculation of the OC conversion rate in premenopausal women. This was to reduce selection bias from including premenopausal women undergoing surgery for a suspicious ovarian mass, as these women would automatically be classified as being at higher risk of OC than those referred for symptoms and abnormal testing alone. In total, 1124 women, including 548 premenopausal and 576 postmenopausal women, were recruited prior to the protocol change.

### 2.5 Timelines

#### 2.5.1 Recruitment

Women completed a baseline questionnaire at recruitment. This usually coincides with a referral by their GP to see a gynaecologist in hospital.

#### 2.5.2 Three months

Most women would have been triaged into low or high risk of OC using the current standard of triage in the UK, which is the Risk of Malignancy Index (RMI) at 3 months.^23^ Women in the high-risk category undergo investigations (biopsies) or interventions (surgery) to attain a histological diagnosis.

#### 2.5.3 Twelve months

Women without a histological diagnosis (no biopsy or surgery at 3 months) were followed up at 12 months. This cohort represents women who tested positive for initial tests (CA125, symptoms indicating possible OC, abnormal imaging) but in whom an OC diagnosis was not confirmed following diagnostic testing or those who were triaged as ‘low risk’ by the RMI tool.

### 2.6 Study measures

All participants completed the Impact of Events Scale – Revised (IES-r) questionnaire and a six-item short form of the State–Trait Anxiety Inventory (STAI-6) at recruitment. For women who had not undergone surgery or biopsy within 3 months of recruitment, questionnaires were administered again at 12 months (Figure S1). When comparing the trajectory in anxiety and distress levels at recruitment and after 12 months, we excluded all participants diagnosed with OC or other cancers during the 12 month period. These two questionnaires have previously been used in cancer screening studies and are validated tools to measure anxiety and distress.

#### 2.6.1 Anxiety

The STAI is a self-assessment questionnaire consisting of 20 items.^24^ Each statement is rated on a four-point scale. Scores range from 20 to 80, with lower scores reflecting milder degrees of anxiety. Scores can be grouped into three clinical categories: ‘no or low anxiety’ (20–37), ‘moderate anxiety’ (38–44) and ‘high anxiety’ (45–80). The shortened STAI-6 used in ROCkeTS is an abbreviated six-item short form of the STAI questionnaire giving a score range from six to 24. To create scores and categories compatible with the original STAI scores, the score for each participant was calculated by dividing their score by six and then multiplying by 20, in accordance with the literature.^25^ This questionnaire was selected as it is quicker for participants to complete in an outpatient setting.

#### 2.6.2 Distress

The IES-r is a set of 22 five-point Likert scale questions to measure distress and yields a total score of 0–88.^26^ Using this tool, participants report on the effects of intrusive thoughts related to an event (i.e. their referral for OC testing) and their efforts to avoid any recollection of this event. Scores on the IES-r are used to define three clinical categories: post-traumatic stress disorder (PTSD) is a clinical concern (24–32); probable PTSD (33–36); and severe enough to suppress the immune system – effects may persist for 10 years following the event (37 or more).

### 2.7 Statistical analysis

The aim of this study was to compare anxiety and distress levels at recruitment for all participants and at 12 months post-testing in women not diagnosed with OC. We also calculated the OC conversion rate in the referred women. Women were grouped as postmenopausal if they had not had a period for over 12 months. In women who had been amenorrhoeic for over 12 months for reasons such as contraception or hysterectomy, menopausal status was assigned according to their age: women up to the age of 50 years were considered premenopausal, whereas those aged 50 years and older were considered postmenopausal. Only women recruited prior to the implementation of protocol version 7.0 were included in the analysis of premenopausal women for this study. This article includes the majority of recruits to the ROCkeTS study. All analyses were performed using Stata 16 (StataCorp LLC,College Station, TX, USA).

#### 2.7.1 Analysis of psychological questionnaires

Categorical data were presented using frequencies and percentages. Scores from the STAI-6 and IES-r questionnaires were treated as continuous variables. The normality of their distributions was assessed. Outcome measures that followed a normal distribution were presented as mean and standard deviation, and as median and interquartile ranges for those with a non-normal distribution. The association between STAI-6 and IES-r scores with explanatory variables was explored using the appropriate parametric or non-parametric tests (Wilcoxon rank-sum test and Kruskal–Wallis test). Results with *P* < 0.05 were considered statistically significant.

#### 2.7.2 Analysis of cancer conversion rates

The OC conversion rate as described by the National Cancer Intelligence Network (NCIN) refers to the percentage of women diagnosed with OC following an urgent referral for suspected OC. We calculated the OC conversion rate in all postmenopausal women recruited and in the subset of premenopausal women recruited prior to the protocol change, which limited the recruitment of premenopausal women to pre-surgical patients only.

## 3 Results

A total of 2596 participants were included in the analysis: 85.1% completed the STAI-6 (2208/2596) and 85.6% completed the IES-r (2222/2596) questionnaires at recruitment (Figure S2); 31.8% (825/2596) had not had a biopsy or undergone surgery at 3 months and received a follow-up questionnaire at 12 months, and 56.6% and 57.3% of these women completed the STAI-6 (467/825) and IES-r (473/825) questionnaires (Figure S2).

The sociodemographic characteristics of all participants are presented in Table 1. The median and interquartile range for age was 53 (43–65) years and 55.2% (1432/2596) were postmenopausal. In this cohort 52.3% (1358/2596) were employed and 45.6% (1185/2596) had at least secondary school level qualifications, 62.6% (1624/2596) lived with a partner and 92.1% (2391/2596) of the participants were white.

The clinical characteristics and outcomes for all women are shown in Table 2: 67.0% (1741/2596) presented via the fast-track pathway; 17.8% (463/2596) were referred by cancer units and other specialties; 8.8% (229/2596) were referred via routine GP referrals; and only 6.4% (163/2596) were referred via the emergency route. Out of all participants, 94.5% (2454/2596) were of good performance status (0 and 1), 43.0% (1117/2596) were current or ex-smokers, 38.2% (991/2596) completed questions about their gynaecological history, 6.0% (156/2596) had a previous history of subfertility, 8.4% (217/2596) reported postmenopausal bleeding, 12.4% (322/2596) had used contraception, 16.7% (436/2596) used hormone replacement therapy and 21.3% (553/2596) had experienced a change in the nature of their periods. Ten women who were initially triaged as being at low risk of OC at 3 months were eventually diagnosed with primary OC at 12 months; 30/2596 women (1.2%) were diagnosed with non-OC at 12 months and breast cancer was the most common non-OC diagnosis.

At recruitment, distress levels were severe in 53.3% (1185/2222), moderate in 7.7% (170/2222) and mild in 39.0% (867/2222), whereas anxiety levels were severe in 48.5% (1071/2208), moderate in 32.2% (710/2208) and mild in 19.3% (427/2208). We compared the median STAI-6 and IES-r scores at recruitment between women who completed the 12-month questionnaire and those who did not. There was no clinical or statistically significant difference in the median STAI-6 scores among responders and non-responders: 43 (40–50) versus 43 (40–50) (*P* = 0.470). Similarly, we did not find any significant difference in the median IES-r scores: 34 (25–53) versus 34 (24–48) (*P* = 0.323).

An analysis of the factors associated with anxiety or distress at recruitment is illustrated in Table 3.

### 3.1 Age and other clinicodemographic variables

Women aged <50 years displayed a higher median (interquartile range) IES-r score compared with women aged ≥50 years: 44 (30–63) versus 40 (28–60) (*P* < 0.00). Being retired was a protective factor for distress, as these participants reported ‘moderate distress’, scoring 36 (27–55), compared with ‘severe distress’ in the rest of the cohort. There was a positive correlation between the level of education and IES-r scores at recruitment. This did not translate to a change in the clinical categories of distress levels, however, as the median IES-r scores were over 37 (i.e. ‘severe’ distress) for women from each education level category. Women who described a change in their periods scored higher on the IES-r questionnaire. There were no correlations between distress levels and clinico-demographic factors (marital status, ethnicity or performance status) or other gynaecological variables (history of subfertility, history of ovarian stimulation, use of contraception, postmenopausal bleeding, use of hormone replacement therapy or parity). There was no clinical or statistical correlation between any of the variables considered and anxiety levels at recruitment.

### 3.2 Route of referral

Routine GP referrals were associated with moderate levels of distress at recruitment, whereas emergency presentations resulted in the highest distress levels: 33 (26–53) versus 45 (30–65) (*P* < 0.00).

**Figure 1:**
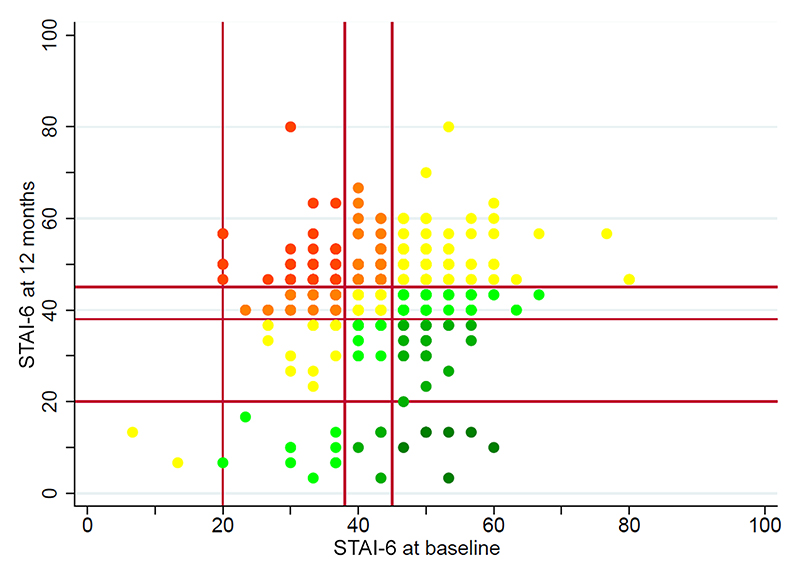
Heat map to illustrate change in STAI-6 scores at recruitment and at the 12 month follow-up among 467 respondents. Red dots represent subjects in whom distress levels became more severe by three categories, yellow in those in whom clinical anxiety or distress category remained unchanged and dark green in those in whom the anxiety or distress levels improved by three categories.

**Figure 2:**
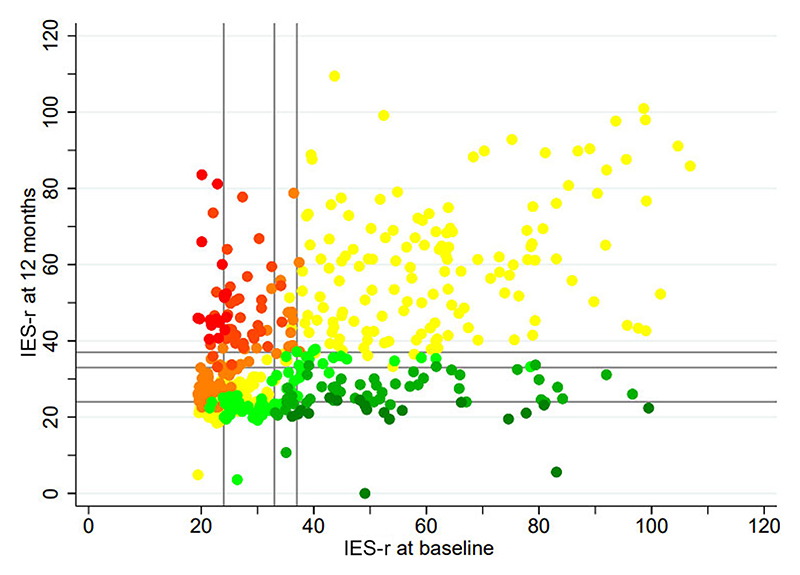
Heat map to illustrate change in IES-r scores at recruitment and at the 12 month follow-up among 473 respondents. Red dots represent subjects in whom distress levels became more severe by three categories, yellow in those in whom clinical anxiety or distress category remained unchanged and dark green in those in whom the anxiety or distress levels improved by three categories.

There was no change in the clinical category for anxiety levels in 46% of respondents, whereas 30% experienced increased anxiety and 24% had improved anxiety by 12 months (Figure 1; Table S1). There was no change in the clinical category for distress levels in 66% of respondents, whereas 20% reported an improvement and 14% experienced more severe distress at 12 months (Figure 2; Table S2).

Overall, the true positive diagnosis rate of OC in premenopausal women was 19/548 (3.5%), compared with 232/1432 (16.2%) for postmenopausal women. Of those referred via the fast-track pathway, 12/363 (3.3%, 95% CI 2.2%–5.4%) premenopausal women were diagnosed with primary OC, compared with 181/979 (18.5%, 95% CI 14.4–18.2) postmenopausal women, at 12 months (Table 4).

The tabulation of OC rates showed that no women referred under the age of 29 years were diagnosed with OC (Table S3). Only 1.6% of women aged <40 years were diagnosed with OC.

## 4 Discussion

### 4.1 Main findings

This large, multicentre, prospective study investigated the psychological impact of diagnostic testing for suspected OC. Most women experience severe distress and anxiety at recruitment, and these remain unchanged/worsened in the majority at 12 months, even in the absence of an OC diagnosis. Our results showed that women aged ≥50 years reported lower distress compared with women aged <50 years. Women who presented via the emergency pathway reported the highest level of distress. The OC conversion rate was at least four times higher in postmenopausal women compared with premenopausal women. The OC conversion rate varied substantially by age, with very low rates in women aged <40 years (1.6%, 95% CI 0.5%–5.9%) compared with women aged ≥40 years (10.9%, 95% CI 8.7%–13.6%). In summary, younger women display higher levels of distress but are much less likely to receive a diagnosis of OC. Their persistent high anxiety levels despite not being diagnosed with OC suggest that they mandate additional support. To the best of our knowledge, our study is the first multicentre prospective study including outcomes for women referred under the fast-track pathway.

### 4.2 Interpretation, in light of other evidence

Our results support findings from screening studies that described a significant increase in OC-specific worry and distress even in women without a cancer diagnosis.^27–33^ A recent study by Lof et al. explored the experiences of a referral and workup for surgery for an ovarian mass.^17^ Their results demonstrated that 57% of recruits experienced clinically significant cancer-specific distress levels preoperatively when the histology was unclear. Overall, 99% of women who were identified with a benign ovarian mass were satisfied with the diagnostic pathway. The authors therefore concluded that patients were receptive to a referral for investigations and treatment, even if their estimated risk of OC was low. Our study differs from that of Lof et al. in a number of ways. Our participants were not restricted to those scheduled for surgery only. We also compared anxiety and distress levels at two time points to accurately assess the detrimental effects of a referral for possible OC, especially in women with a false-positive result.

Our results illustrate that there is a clear association between a referral for testing for OC and high anxiety and distress levels, which remain elevated at 12 months even in the absence of an OC diagnosis. National statistics demonstrate that one in three adults experience high anxiety levels and that 30% of the population experiences some form of distress, with 6.2% reporting ‘high’ levels.^34^ Approximately one in two women recruited to our study reported high levels of anxiety and distress at recruitment, i.e. higher than UK baseline rates. It is possible that our participants represent the ‘worried well’, that is women who perceive themselves as being more likely to develop OC and have a higher awareness of the manifestations of OC, and therefore present to their GPs with these symptoms. These are usually educated patients who are often proficient at information seeking.^35,36^

Anxiety levels remained unchanged or worsened in 76% of women despite the absence of an OC diagnosis at 12 months. This could be explained by a higher level of trait anxiety, as suggested by Wiggins et al.^37^ The authors measured OC-specific distress levels at various time points (baseline, 1 month and 4 months) in 373 women recalled following a false-positive transvaginal ultrasound scan (TVS) result. Their results demonstrated that although distress levels declined in women with high baseline levels, these remained elevated at 4 months. A multivariate analysis identified a family history of OC, a monitoring coping style and weaker optimism as possible predisposing factors for distress. Our study further demonstrates a negative correlation between age and distress levels. Lower anxiety levels in older women have also been noted in the UK Collaborative Trial of Ovarian Cancer Screening (UKCTOCS).^32^

### 4.3 Strengths and limitations

ROCkeTs is a prospective study and our analysis includes a sample of over 2500 women recruited from 24 sites. For patients with missing data, additional information was procured by the research nurses through accessing the medical records or by contacting their GPs. Our protocol was also carefully designed to exclude all those with a diagnosis of cancer at 12 months as part of our pre-planned analysis plan. Our inclusion criteria were restricted to newly presenting patients to ensure that our cohort rigorously adhered to the criteria for OC testing. Finally, this is the first multicentre study that explores the OC conversion rate in premenopausal and postmenopausal women referred to fast-track clinics. Our finding of differential OC rates by age is consistent with the epidemiology of OC, with a peak incidence rate for women in their 70s.^38^

One of the limitations of our study is that the majority of responders were white females. We concede that women who declined or withdrew consent may either have been over-whelmed or dissuaded from taking part following language, cultural or socio-economic obstacles. Our results may therefore not be representative of some of the most vulnerable subgroups of women. However, we did not find a significant difference in anxiety and distress levels at recruitment among responders and non-responders at 12 months. This suggests that factors other than higher levels of pre-existing anxiety and distress should be considered to account for the loss to follow-up rate in this study. Follow-up data were only available for 60% of women without a diagnosis of cancer at 12 months and may not be representative of all women in this category.

It was not possible to infer causality or demonstrate whether abnormal preliminary results and a referral to hospital for further testing generate higher anxiety and distress. First, a comparison of the anxiety and distress levels among women with a true negative result (symptomatic but normal results) and those with a false-positive result (symptomatic and abnormal results) would be necessary to explore to what degree the presence of symptoms and a fear of cancer contribute to anxiety and distress. Second, we do not have a measure of baseline anxiety and distress levels in women in the community. It was therefore not possible to demonstrate a temporal relationship between a referral for OC testing and a change in anxiety and distress levels.

### 4.4 Implications for practice and future research

Our results also accentuate an urgent need to review current practice to implement an age-stratified referral pathway. In this study, the prevalence of OC in women aged <50 years did not reach the 3% threshold advocated by the National Institute for Health and Care Excellence (NICE). Funston et al. demonstrated that a higher CA125 threshold of 89 U/mL is necessary to attain a 3% probability in women aged <50 years, compared with 39 U/mL for those aged ≥50 years, and proposed that clinicians should be more selective in referring patients for further investigations (i.e. those with a risk of >3% calculated from their age and CA125).^39^ Alternatively, women could be counselled about their individualised risk of OC and a plan of care mutually agreed. Current international guidelines do not place an emphasis on OC testing by age or menopausal status.^40–42^ Further quantitative research accompanied by qualitative research is warranted to evaluate the effectiveness and patient experience of any interventions to mitigate the adverse psychological impact of testing in this population.

Efforts should focus on diagnostic pathways such as one-stop clinics to ensure a rapid diagnosis and address the fragmentation across multiple appointments, as well as improving psychological outcomes.^43,44^ There is evidence to support its use in reducing anxiety in breast cancer testing.^44,45^ Future research focusing on women deemed to be at low risk, e.g. under the age of 40 years, is essential to investigate how anxiety and distress levels compare among women referred via different routes, explore what is needed/wanted by women to improve their experience of the OC testing pathway and evaluate whether the fast-track pathway contributes to higher psychological morbidity in this cohort, or whether it mitigates these by reducing waiting times.

## 5 Conclusion

This study demonstrates that women under the age of 50 years and those who were referred via the emergency route are at high risk of distress. Patients referred to gynaecology clinics with an abnormal CA125 level and/or ultrasound tests for OC testing have high persistent levels of anxiety and distress, irrespective of the final histological diagnosis. Efforts should focus on improving counselling and support in young women, especially for women under the age of 40 years, as the prevalence of cancer in these women is 1.6% which is below the NICE recommended threshold of 3% for cancer testing. Considering the increase in anxiety and distress demonstrated by our study in women under 50 years of age, combined with the prevalence of OC in women under 40 years of age, we would suggest that the harms of testing for OC in women under 40 years of age outweigh the benefits.

## Supplementary Material

Additional supporting information can be found online in the Supporting Information section at the end of this article.

Appendix

Figure S1

Figure S2

Table S1

Table S2

Table S3

## Figures and Tables

**Table 1:** Sociodemographic characteristics of all participants.

Characteristic	Number (%)^a^ *N* = 2596
Age	
Median (interquartile range), years	53 (43–65)
Postmenopausal	
Yes	1432 (55)
No	1164 (45)
Living situation	
Lives alone	875 (34)
Lives with partner	1624 (62)
Unknown	97(4)
Employment status	
Employed	1202 (46)
Self-employed	156 (6)
Retired	790 (30)
Unemployed	169 (7)
Student or other	186 (7)
Unknown	93 (4)
Highest level of education	
No qualifications	504 (19)
At least secondary school level	1185 (45)
At least tertiary level	598 (23)
Other	209 (8)
Unknown	100 (4)
Ethnic group	
White	2311 (89)
Non-White	192 (7)
Unknown	93 (4)

a Unless otherwise stated.

**Table 2:** Clinical characteristics and outcomes of participants.

Characteristic	*N* (%) *N =* 2596
Route of presentation	
Accident and emergency	163 (6)
Fast-track pathway	1741 (67)
Referral from cancer unit or cross-specialty referral	463 (18)
Routine GP referral	229 (9)
World Health Organization performance status	
0	2178 (83)
1	276 (11)
2	65 (3)
3	34 (1)
4	2 (<1)
Unknown	41 (2)
Ever smoked	
Yes	1117 (43)
No	1384 (53)
Unknown	95 (4)
History of subfertility	
Yes	156 (6)
No	839 (32)
Unknown	1601 (62)
Change in nature of periods	
Yes	553 (21)
No	442 (17)
Unknown	1601 (62)
Past or current use of contraception	
Yes	322 (13)
No	669 (26)
Unknown	1605 (62)
History of postmenopausal bleeding	
Yes	217 (8)
No	742 (29)
Unknown	1637 (63)
Past or current use of hormone replacement therapy	
Yes	436 (17)
No	1039 (40)
Unknown	1121 (43)
Clinical outcomes	
Diagnosed with primary OC	251 (10)
Diagnosed with non-OC at 12 months	30 (1.2)^a^
Breast	7 (23)
Uterine	5 (17)
Bowel	5 (17)
Lymphoma	3 (10)
Lung	3 (10)
Renal	3 (10)
Gastric	2 (7)
Pancreatic	1 (3)
Skin	1 (3)

a *n* (% of cases of non-OC).

**Table 3:** Analysis of factors associated with anxiety or distress at recruitment.

	Responses*n* (%)	STAI-6 score Median (IQR)	IES-r score Median (IQR)
Age^a,b^		*P*= 0.82	***P*< 0.00**
<50 years	989 (38)	43 (40–50)	44 (30–63)
≥50 years	1606 (62)	43 (40–50)	40 (28–60)
Marital status^b,c^		*P*=0.71	*P*= 0.69
Living alone	875 (35)	43 (40–50)	42 (29–60)
Living together	1624 (65)	43 (40–50)	41 (29–63)
Other	2 (2)	48 (43–53)	39 (28–49)
Employment status^b,c^		*P*= 0.08	***P*< 0.00**
Employed full-time or part-time	1202 (48)	43 (40–50)	44 (30–63)
Self-employed	156 (6)	43 (40–50)	41 (28–59)
Retired	790 (32)	43 (40–50)	36 (27–55)
Unemployed	169 (6)	47 (40–50)	43 (29–72)
Student	186 (8)	47 (40–50)	42 (28–64)
Education level^b,c^		*P*= 0.21	***P*** = **0.00**
No qualifications	504 (20)	47 (40–50)	38 (27–59)
At least secondary level	1185 (47)	43 (40–50)	42 (29–63)
At least tertiary level	598 (24)	43 (40–50)	44 (31–62)
Other	209 (9)	43 (40–50)	38 (26–59)
Ethnicity^a,b^		*P*= 0.20	*P*= 0.27
Non-white	192 (8)	47 (40–50)	42 (29–70)
White	2311 (92)	43 (40–50)	41 (29–61)
Ever smoked^a,b^		*P*= 0.85	***P*=0.03**
No	1384 (55)	43 (40–50)	41 (28–60)
Yes	1117 (45)	43 (40–50)	42 (30–63)
Route of presentation^b,6^		*P*= 0.62	***P*< 0.00**
Accident and emergency	163 (6)	47 (40–50)	45 (30–65)
Rapid-access clinic referrals	1741 (67)	43 (40–50)	43 (30–63)
Cancer unit or other specialty	463 (18)	43 (40–50)	39 (27–59)
Routine GP referral	229 (9)	43 (40–50)	33 (26–53)
Performance status^c^		*P*= 0.64	*P*= 0.61
0	2178 (85)	43 (40–50)	42 (29–62)
1	276 (11)	47 (40–50)	40 (29–60)
2	65 (3)	43 (40–50)	39 (29–62)
3	34 (1)	43 (40–50)	35 (28–48)
4	2 (<1)	45 (40–50)	42 (38–46)
History of subfertility^a^		*P*= 0.55	*P*= 0.88
No	839 (84)	43 (40–50)	45 (31–63)
Yes	156 (16)	43 (40–50)	47 (30–60)
History of ovarian stimulation for subfertility^a^		*P*= 0.10	*P*= 0.84
No	927 (93)	43 (40–50)	45 (31–63)
Yes	65 (7)	47 (40–50)	48 (32–62)
Change in nature of periods^a^		*P*= 0.25	***P*= 0.02**
No	442 (44)	43 (40–50)	44 (29–61)
Yes	553 (56)	43 (40–50)	47 (32–64)
Use of contraception^a^		*P*= 0.96	*P*= 0.30
No	669 (67)	43 (40–50)	46 (31–64)
Yes	332 (33)	43 (40–50)	44 (30–61)
Postmenopausal bleeding^a^		P = 0.50	P = 0.89
No	742 (77)	47 (40–50)	38 (28–60)
Yes	217 (23)	43 (40–50)	38 (28–60)
Current of previous use of hormone replacement therapy^a^		P = 0.08	P = 0.17
No	1039 (70)	47 (40–50)	38 (28–59)
Yes	436 (30)	43 (40–50)	40 (29–61)
Number of pregnancies^c^		P = 0.78	P = 0.15
0	97 (5)	43 (40–50)	43 (32–65)
1–4	1645 (91)	43 (40–50)	41 (29–61)
5 or more	61 (4)	47 (40–50)	34 (24–66)

Bold denotes values which are statistically significant i.e. *P*<0.05.

a The Wilcoxon rank-sum test was used to calculate the P-value.

b Variables identified as part of a systematic review.

c The Kruskall–Wallis test was used to calculate the *P*-value.

**Table 4:** OC conversion rate per mode per mode of presentation in premenopausal women prior to protocol change^a^ and all post-menopausal women.

	Premenopausal				Postmenopausal			
	Diagnosed with OC	Referred	% (95% CI)		Diagnosed with OC	Referred	% (95% CI)	
Accident and emergency	3	32	9.4 (3.2, 24.2)		11	67	16.4 (9.4, 27.0)	
Fast-track pathway	12	363	3.3 (1.9, 5.7)		181	979	18.5 (16.1, 21.0)	
Cancer unit or cross-specialties	2	77	2.6 (0.7, 9.0)		36	290	12.4 (9.1, 16.7)	
Routine GP referral	2	76	2.6 (0.7, 9.1)		4	96	4.2 (1.6, 10.2)	
Overall	19	548	3.5 (2.2, 5.4)		232	1432	16.2 (14.4, 18.2)	

*Note*: % represents the proportion of women presenting via a mode of presentation and who were identified with a true diagnosis of OC.

a In pre-menopausal women, protocol for recruitment was altered in 2018 to only include women undergoing surgery or biopsy due to the very low rate of cancer in recruited participants.

## Data Availability

The data that support the findings of this study are available from the corresponding author, upon reasonable request. Subject to data sharing agreements and approval by the Project oversight group.
