# Supplementary material for: Investigating harms of testing for ovarian cancer – psychological outcomes and cancer conversion rates in women with symptoms of ovarian cancer: A cohort study embedded in the multicentre ROCkeTS prospective diagnostic study
Source: BJOG. Author manuscript; Available in PMC 2024 Sep 1. (PMC7616335; doi:10.1111/1471-0528.17813)
Supplement: Appendix [file EMS195168-supplement-Appendix.pdf]

Appendix 1. ROCkeTS collaborators

Regional Study Centre Team

Belfast City Hospital
Nagar H (PI & imaging lead), McAlister C, Clarke, P, O'Donnell A, Cunningham W, McAllister J, McClisker S, McClean S, Dadebo B, Laverly G

Birmingham City Hospital
Sundar S (PI), Parker R (Associate PI), Khan H (Imaging lead), Butler L, Gammon B, Samuel-Oparah U, Orme A, Marsden L, Smith G, Cartwright J, Storistreet D, Goddard H, Williams A, Bruten E, Devonport D, Pilsworth Z

Birmingham Women's Hospital
Abedin P (PI), Balogun M (Imaging lead), McCooty S, Qureshi N, Chana P, Beale F, Galloway A, Iqbal G, Carden N, McPake C

East Surrey Hospital
Jermy K (PI & Imaging lead), Weller S, Maher S, Summers G, Nicks H, Knight H, Habibi R

Guy's Hospital
Sayasheh A (PI and Imaging lead), Abdelbar A (Associate PI), Debattista L, D'Alessandro V, Bilbert-Jones H, Khaula M, Ijeomah-Orgi M, Worthington M, Fitzpatrick-Greening M, Lombardi S, Ng L, Shipa B, Zielonka A, Jadhau A, Barrett S, Love R, Borley J, Mohamoud N

Hinchingbrooke Hospital
Majmudar T (PI & Imaging lead), Mackenzie C (Associate PI), Palombo C, Baker TA, Adebayo A, Wilde L, Nosib H, Miller S, Webb D, Perkins L, Plaza S, Goss V, Donnelly S, Osmanska A, Kurian R, Lam R, Calcada R, Marco-Illana E

James Cook University Hospital
Hebblethwaite N (PI), Exley K (Imaging lead), Peatman S, Kane J, Hebbron K, Alexander H, Harwood H, Cuthbert H, Hodges M, McNeil J, Wright L, Dale M, Chadwick V, Naseem S, Iqbal N, Proctor C

Liverpool Women's Hospital
McDonald RD (PI), Hamer M (Imaging lead), Robinson-Jones A, Pearritt S, Corlett P, Wray J, Drury J, Heathcote L, Sutton V, Coppin D, Cooke K, Bolderson J, Bia C, Sawan S, Davies M, Lowe A, Hamlett H, Houghton F, Beasley A, Robinson-Jones A, Rice E, Bell S

Norfolk & Norwich University Hospital
Duncan T (PI), Ames V (Imaging lead), Archer D, Gibbins T, Turner S, Nieto J, Borbos N, Turnbull H, Anderson S, French K, Hunter N, High L, Dann A, Licence V, Websdale C, Darby H, Malone E, Walton S, Schofield E, Platt J, Cooper A, Cook J, Cornwell M, Ashgar M, Walter S, Macnab W, Kellett J, Halliwell-Bass S, Knapp S, McElhinney S

Northampton General Hospital
Gnanachandran C (PI & Imaging lead), Alawad H (Associate PI), Kariyadil B, Jose S, Kempa A, Woolhouse C, Duncan A, Bussey R, Campey L, Hall K, Dudgeon L, Hitchcock R, Polnik M, Stockham LJ, Al Husain H, Grantham G

Nottingham City Hospital

Gajjar K (PI & Imaging lead), Coleridge S/ Naskretski A (Associate PI), Dennis S, Gibbins T, Williamson K, Nunns D, Abu J, Hammond R, Juliana A, Golding J, Cope J, Mills S, Gan C, Wrigmy S, Warren C, Ward H, Wilson G

Peterborough General Hosptial
Ramsay B (PI), Moshy R (Imaging lead), Adebayo A, Palombo C, Woodhouse S, Barter E, Baker TA, Butcher D, Goodyear P, O'Sullivan S, O'Herlihy S, Collins H, Sidlow J, Weatherburn A, Steachan S, Diaz S, Austin M, Penart-Buck F, Dunn S, Adams L, Bhayani J

Princess Anne Hospital
Rosello N (PI), Johnson S (Imaging lead), Benson L, Wood J, Lowry J, Smith L, Barton S

Queen Elizabeth Hospital, Gateshead
Hughes T (PI & Imaging lead), Pearce L, McCormick W

Royal Blackburn Hospital
Willett M (PI)

Royal Hallamshire Hospital
Abdi S (PI & Imaging lead), Duffy S, Bullivant E, Taylor F, Waller C, Jobling N, Tidy J, Palmer J, Gillespie A, Senbeto S, Sutcliffe A, Johnson K, Murtagh L, Lally B

Royal Victoria Infirmary
Russell M (PI), Maddison J (Imaging lead), Kimber A, Graham J, Conner D, Murtha V, Dunn E, Lim CP, Russell M, Chalhoub T, Birtles JO, Davies M, Galeon MC, Lowes J, Narayansingh G, Fenn A, Gallgher I, Brown K, Hoh J

The Royal London Hospital St Bartholomew's Hospital
Manchanda R (PI & Imaging lead), Aswat S, Robbani S, Dzumbunu F, Chandrasekaran D, Gaba F, Lawrence A, Sahdev A, Hillman P

Royal Sussex County Hospital
Kaushik S (PI), Baron S (Associate PI), Vitta L (Imaging lead), Herbertson R, Lyttle AJ, Laltho R, Larsen-Disney P, Newman N, Curry J, Heron H, Porges A, McLennan C, Frattaroli p, Temegan J, O'Neill F, Whitfield C, Lavender P, , Dailey N, Drews F, Langford K, Fellich V

Royal Preston Hospital
Keating P/Wood N (PI), Butcher T (Imaging lead), Young A, Cornthwaite S, Swan A, Martyniak A, Brunton M, Sutton V, Turner E, Ellel K, Antrobus P, Leach M, Musa N, Ardern N, Prashar S, Jones K, Brar N, Cook A, Patel S, Gardner A, Panchal K, Speirs R

University Hospital of North Durham
Sengupta P (PI), Kent R (Imaging lead), Deur J, Downey L, Sen S, Atkinson V, Bodnar S, wook M, Walton E, Arava U, Rathaparchi S, Damigos E, Kay A, Potts K, Chatt R, Jennings J, Baggett A, Beukenholdt R, Bainbridge V, Clark S, Nemeth Z, Humphries C, Stamp K, Brown E, O'Brien J, Hobson S

Sheffield Teaching Hospitals
Palmer J (Imaging lead)

University Hospital of Wales
Sharma A (PI), Sinha A (Imaging lead), Noble N, Wadmore C, Holland R, Pugh N, Lim K, Rzyska E, Shamsunsin L, Gnanachandrana C, Kendall J, Price C, Cloudsdale R, McNee D, Smith C, Heirene L, James R

Walsall Manor Hospital
Ghazal F (PI), Rai H (Imaging lead), Davies J, Mhembere P, Botfield L, Fletcher J, Darby S, Lenehan F, Richardson L, Thomas T, Hannon J, Cogings P

Watford General Hospital
Radhikavikram S (PI), Malhotra V (Imaging lead), Walker E, Markwell K, Zhao X
