# Supplementary material for: Investigating harms of testing for ovarian cancer – psychological outcomes and cancer conversion rates in women with symptoms of ovarian cancer: A cohort study embedded in the multicentre ROCkeTS prospective diagnostic study
Source: BJOG. Author manuscript; Available in PMC 2024 Sep 1. (PMC7616335; doi:10.1111/1471-0528.17813)
Supplement: Figure S1 [file EMS195168-supplement-Figure_S1.docx]

**S1 Fig. Study design flowchart**

** These two questionnaires include the STAI-6 and IES-r tools.*

**Baseline questionnaire***

**Participation complete**

**Outcome questionnaire***

Did the participant undergo surgery or biopsy within 3 months

Yes

No

Recruitment

3 months

12 months
