# Supplementary material for: Investigating harms of testing for ovarian cancer – psychological outcomes and cancer conversion rates in women with symptoms of ovarian cancer: A cohort study embedded in the multicentre ROCkeTS prospective diagnostic study
Source: BJOG. Author manuscript; Available in PMC 2024 Sep 1. (PMC7616335; doi:10.1111/1471-0528.17813)
Supplement: Figure S2 [file EMS195168-supplement-Figure_S2.docx]

**S2 Fig. Recruitment flowchart**

#

350 did not respond

342 did not respond

388 did not respond

374 did not respond

Participation complete

**12 months**

**3 months**

**Recruitment**

475 completed STAI-6 questionnaire

483 completed IES-r questionnaire

825 women sent IES-r and STAI-6 questionnaires

1171 underwent surgery and/or biopsy

- Primary cancer – 379, inc 279 OC
- Secondary cancer – 30
- Borderline tumour – 126
- Neoplasm of uncertain behaviour – 6
- Benign/normal – 1176
- Other – 50
- NA - 4

825 did not have surgery or a biopsy

2222 completed IES-r questionnaire

2208 completed STAI-6 questionnaire

2596 women recruited to ROCkeTS and sent IES-r and STAI-6 questionnaires
