# Supplementary material for: Investigating harms of testing for ovarian cancer – psychological outcomes and cancer conversion rates in women with symptoms of ovarian cancer: A cohort study embedded in the multicentre ROCkeTS prospective diagnostic study
Source: BJOG. Author manuscript; Available in PMC 2024 Sep 1. (PMC7616335; doi:10.1111/1471-0528.17813)
Supplement: Table S1 [file EMS195168-supplement-Table_S1.docx]

**S1 Table.** Tabulation of change in STAI scores at recruitment and 12 months follow up among 467 respondents

|  | | **Anxiety level at 12 months n (%)** | | | |
| --- | --- | --- | --- | --- | --- |
|  |  | **Mild** | **Moderate** | **Severe** | **Total** |
| **Anxiety level at recruitment** | **Mild** | 29 (6) | 32 (7) | 44 (9) | 105 |
|  | **Moderate** | 21 (4) | 44 (10) | 67 (14) | 132 |
|  | **Severe** | 32 (7) | 60 (13) | 138 (30) | 230 |
|  | **Total** | 82 | 136 | 249 | 467 |

Colour codes:

No change in clinical category

Improved by one clinical category

Improved by two clinical categories

Worsened by one clinical category

Worsened by two clinical categories
