# Supplementary material for: Investigating harms of testing for ovarian cancer – psychological outcomes and cancer conversion rates in women with symptoms of ovarian cancer: A cohort study embedded in the multicentre ROCkeTS prospective diagnostic study
Source: BJOG. Author manuscript; Available in PMC 2024 Sep 1. (PMC7616335; doi:10.1111/1471-0528.17813)
Supplement: Table S2 [file EMS195168-supplement-Table_S2.docx]

**S2 Table.** Tabulation of change in IES-r scores at recruitment and 12 months follow up among 473 respondents

|  | | Distress level at 12 months n (%) | | | |
| --- | --- | --- | --- | --- | --- |
|  |  | Mild | Moderate | Severe | **Total** |
| Distress level at recruitment | Mild | 159 (34) | 11 (2) | 50 (10) | 220 |
|  | Moderate | 18 (4) | 3 (1) | 12 (2) | 33 |
|  | Severe | 61 (13) | 16 (3) | 143 (31) | 220 |
|  | **Total** | 238 | 30 | 205 | **473** |

Colour codes:

No change in clinical category

Improved by one clinical category

Improved by two clinical categories

Worsened by one clinical category

Worsened by two clinical categories
