# Supplementary material for: Investigating harms of testing for ovarian cancer – psychological outcomes and cancer conversion rates in women with symptoms of ovarian cancer: A cohort study embedded in the multicentre ROCkeTS prospective diagnostic study
Source: BJOG. Author manuscript; Available in PMC 2024 Sep 1. (PMC7616335; doi:10.1111/1471-0528.17813)
Supplement: Table S3 [file EMS195168-supplement-Table_S3.docx]

**S3 Table.** Incidence of OC by age group in fast-track referrals only in all postmenopausal and women and in premenopausal women prior to protocol change

| **Age group** | **n*** | **N**** | **% (95% CI)** |
| --- | --- | --- | --- |
| **Under 20** | 0 | 8 | 0 (0, 32.4) |
| **20 to 29** | 0 | 42 | 0 (0, 8.4) |
| **30 to 39** | 2 | 70 | 2.9 (0.8, 9.8) |
| **40 to 49** | 5 | 186 | 2.7 (1.2, 6.1) |
| **50 to 59** | 19 | 197 | 9.6 (6.2, 14.6) |
| **60 to 69** | 24 | 139 | 17.3 (11.9, 24.4) |
| **Over 70** | 21 | 111 | 18.9 (12.7, 27.2) |

*n represents number of women with a true diagnosis of OC

**N represents the total number of women referred via rapid access clinics

% represents the proportion of women referred via rapid access clinics who were identified with a true diagnosis of OC
